# Inhibition of Notch Signaling in Combination with Paclitaxel Reduces Platinum-Resistant Ovarian Tumor Growth

**DOI:** 10.3389/fonc.2014.00171

**Published:** 2014-07-07

**Authors:** Jolijn W. Groeneweg, Celeste M. DiGloria, Jing Yuan, William S. Richardson, Whitfield B. Growdon, Sriram Sathyanarayanan, Rosemary Foster, Bo R. Rueda

**Affiliations:** ^1^Vincent Department of Obstetrics and Gynecology, Vincent Center for Reproductive Biology, Massachusetts General Hospital, Boston, MA, USA; ^2^Department of Obstetrics, Gynecology and Reproductive Biology, Harvard Medical School, Boston, MA, USA; ^3^Merck Research Laboratories, Boston, MA, USA; ^4^Division of Gynecologic Oncology, Department of Obstetrics and Gynecology, Massachusetts General Hospital, Boston, MA, USA

**Keywords:** ovarian cancer, Notch, γ-secretase inhibitor, chemoresistance, patient-derived xenograft

## Abstract

**Introduction:** Ovarian cancer (OvCa) is the most lethal gynecologic malignancy in the United States because of chemoresistant recurrent disease. Our objective was to investigate the efficacy of inhibiting the Notch pathway with a γ-secretase inhibitor (GSI) in an OvCa patient-derived xenograft model as a single agent therapy and in combination with standard chemotherapy.

**Methods:** Immunocompromised mice bearing xenografts derived from clinically platinum-sensitive human ovarian serous carcinomas were treated with vehicle, GSI (MRK-003) alone, paclitaxel and carboplatin (P/C) alone, or the combination of GSI and P/C. Mice bearing platinum-resistant xenografts were given GSI with or without paclitaxel. Gene transcript levels of the Notch pathway target *Hes1* were analyzed using RT-PCR. Notch1 and Notch3 protein levels were evaluated. The Wilcoxon rank-sum test was used to assess significance between the different treatment groups.

**Results:** Expression of Notch1 and 3 was variable. GSI alone decreased tumor growth in two of three platinum-sensitive ovarian tumors (*p* < 0.05), as well as in one of three platinum-sensitive tumors (*p* = 0.04). The combination of GSI and paclitaxel was significantly more effective than GSI alone and paclitaxel alone in all platinum-resistant ovarian tumors (all *p* < 0.05). The addition of GSI did not alter the effect of P/C in platinum-sensitive tumors. Interestingly, although the response of each tumor to chronic GSI exposure did not correlate with its endogenous level of Notch expression, GSI did negatively affect Notch signaling in an acute setting.

**Conclusion:** Inhibiting the Notch signaling cascade with a GSI reduces primary human xenograft growth *in vivo*. GSI synergized with conventional cytotoxic chemotherapy only in the platinum-resistant OvCa models with single agent paclitaxel. These findings suggest inhibition of the Notch pathway in concert with taxane therapy may hold promise for treatment of platinum-resistant OvCa.

## Introduction

Ovarian cancer (OvCa) represents the second most common, and most lethal gynecologic malignancy in the United States. By the end of 2013, it is estimated that over 22,000 women will have been diagnosed with OvCa, and more than 15,000 patients will have succumbed to the disease (1). The high death rate is due in part to the fact that the majority of patients present with advanced stage disease possibly due to a lack of screening strategies that effectively detect the cancer in early stages. The most common subtype is high-grade serous carcinoma (70%), followed by endometrioid and clear cell variants (2). Despite the histologic variation, the current treatment regimen includes cytoreductive surgery as well as six cycles of taxane and platinum-based chemotherapy delivered IV or IP (3, 4). While 70–80% of patients respond to this first-line chemotherapy, recurrences occur frequently and a significant majority of those patients develop chemoresistant disease (5, 6). The overall prognosis of patients diagnosed with OvCa therefore remains poor, with a 5-year survival rate of approximately 50% (4). Insights into key molecular pathways that are overexpressed in OvCa and contribute to tumor progression, recurrence, and chemoresistance may lead to novel therapies that can improve the treatment strategies for these women.

The Notch signaling cascade has been implicated in numerous malignancies (7–11) and is one of the most altered pathways in serous OvCa (2). Notch signaling is involved in the regulation of proliferation, differentiation, cell fate, and survival and plays an important role in embryonic development as well as in self-renewal of adult stem/progenitor cells (12–14). Activation of the Notch signaling cascade occurs through binding of one of four Notch receptors (Notch1–4) with one of its ligands (Jagged and Delta-like) on a neighboring cell. Sequential proteolytic cleavage of the Notch receptor by a disintegrin and metalloprotease (ADAM) and γ-secretase leads to the release of the Notch intracellular domain (NICD). This active fragment subsequently translocates to the nucleus, where it activates transcription of target genes including members of the *HES* and *HEY* families (12, 15).

Recent studies have demonstrated expression of the Notch pathway in many malignancies including breast, intestinal, pancreatic, brain, and OvCa (10, 16–21). Genomic analyses of ovarian carcinomas as part of The Cancer Genome Atlas (TCGA) Project showed alteration of Notch signaling in 22% of analyzed tumors (2). In addition, Notch1 and Notch3 RNA transcript and protein are highly expressed in ovarian carcinomas (22–27) and elevated expression correlates with resistance to chemotherapy and decreased survival (28–30). Investigators have hypothesized that Notch signaling may promote the activity of a tumor-initiating cell population that can sustain the growth of ovarian tumors despite cytotoxic therapies that halt the progression of actively proliferating cancer cells (31–33). While this hypothesis is controversial, it would provide rationale for combining Notch pathway inhibition with conventional cytotoxic therapy.

Targeting of the Notch pathway is currently being investigated in a variety of cancers, with γ-secretase inhibitors (GSIs) being the most widely used Notch inhibitors (34, 35). Few phase I and II clinical trials have reported GSI anti-tumor activity though these studies have included only a few women with OvCa. Previous pre-clinical studies have shown that inhibition of Notch signaling blocks the growth of both OvCa cell lines *in vitro* and cell line-derived xenografts *in vivo* (36–38). In addition to inhibition of cell proliferation and induction of cell death, treatment with GSI is associated with an increased sensitivity of OvCa cell lines to platinum therapy (39) supporting the concept that treatment with Notch antagonists may be relevant in a recurrent disease setting (28).

In the current study, we analyzed the contribution of the Notch pathway to the pathobiology of serous OvCa. We assessed the expression of specific members of the Notch family and tested the anti-tumor activity of the GSI MRK-003 against patient-derived xenografts (PDXs) generated from serous carcinomas of the ovary or peritoneum. The pre-clinical efficacy of MRK-003 has been determined in several human cancers (8, 9, 34, 40–43), and we wanted to assess the effectiveness of this GSI as a single agent and in combination with standard cytotoxic chemotherapy in a cohort of clinically platinum-sensitive and resistant ovarian tumors. Treatment of mice harboring the ovarian tumor xenografts with MRK-003 resulted in reduced tumor growth in a subset of experiments. Combination cytotoxic chemotherapy and GSI demonstrated synergistic activity only in those platinum-resistant tumors suggesting Notch pathway inhibition may be more effective in the recurrent and refractory setting.

## Materials and Methods

### Generation and propagation of ovarian cancer xenografts

Our PDX model utilizing primary human ovarian tumors has been described previously (44). Briefly, excess serous OvCa tissue or ascites was obtained at the time of surgery from patients who had given informed consent to participate in an Institutional Review Board approved tissue collection protocol. Following enzymatic processing, tumor-derived single-cell suspensions were depleted of hematopoietic and endothelial cells, resuspended in PBS, mixed with Matrigel (1:1), and injected subcutaneously (s.c.) in female NOD/SCID mice. The range of cells injected was 7.5 × 10^5^ to 1.5 × 10^6^ in each animal. Tumor formation in the injected animals was regularly monitored, and tumors were harvested from euthanized animals when they reached a diameter of 1–2 cm. The harvested xenografts were then enzymatically processed, depleted of H-2Kd positive mouse cells, and injected s.c. into NOD/SCID mice as described above. Using this transplantation system, cohorts of 15–40 mice injected with OvCa cells derived from a single patient were generated and subsequently used to perform treatment experiments.

### Immunohistochemical analysis

Notch1 and Notch3 protein expression in formaldehyde fixed and paraffin embedded sections of primary and xenograft ovarian tumors were analyzed by immunohistochemistry (IHC) using a mouse monoclonal anti-Notch1 antibody (Novus Biologicals) or a rabbit polyclonal anti-Notch3 antibody (Abgent). Non-specific binding of antibody was blocked by either the Vector Laboratories M.O.M. kit (in the case of Notch1) or 5% normal goat serum in PBS with 0.1% Triton X (Notch3) followed by incubation with the relevant primary antibody and the appropriate biotinylated secondary antibody. Subsequent treatment with Vectastain ABC reagents (Vector Laboratories), visualization with 3,3′-diaminobenzidine chromogen (DAB, Dako), and counterstaining with hematoxylin were performed.

### Immunoblotting

Whole cell lysates were prepared from frozen xenograft samples or cell lines using Mammalian Protein Extraction Reagent (Thermo Scientific) lysis buffer supplemented with inhibitors of endogenous protease, kinase, and phosphatase activity (all obtained from Sigma-Aldrich). Twenty micrograms of protein from each sample were separated on 7.5 or 10% polyacrylamide gels and transferred to a PVDF membrane. Following transfer, membranes were blocked with 5% milk in 1× TBS, 0.1% Tween-20 (TBST) and incubated in diluted (1:1000) primary antibody overnight, according to the manufacturer’s recommendations. Primary antibodies used were a rabbit monoclonal anti-Notch1 antibody, a rabbit monoclonal anti-cleaved Notch1 (Val1744) antibody, and a rabbit monoclonal anti-Notch3 antibody (Cell Signaling). Membranes were then incubated with a horseradish peroxidase (HRP) conjugated goat anti-rabbit secondary antibody (Santa Cruz Biotechnology) and developed using a chemiluminescent detection reagent obtained from GE Healthcare Life Sciences. Equivalent protein loading was verified by stripping the blots and re-probing with a mouse anti-Pan-Actin antibody (NeoMarkers).

### Cell culture and *in vitro* treatment with MRK-003

The OVCAR3 and SKOV3 human OvCa cell lines were purchased from ATCC. For MRK-003 dose response experiments, equal numbers of OVCAR3 or SKOV3 cells were plated and serum-starved overnight in growth medium containing 1% FBS. Cells were incubated in triplicate (OVCAR3) or quadruplicate (SKOV3) with either 0, 1, 5, or 10 μM MRK-003. After 48 h, cells were harvested and quantified. Subsequently, OVCAR3 and SKOV3 cells were treated with either the relevant MRK-003 IC_50_ or vehicle control only. Cells were harvested 6 h after administration of MRK-003, and cell pellets were frozen for immunoblotting and quantitative PCR analyses.

### Treatment of mice bearing serous ovarian cancer xenografts

All xenograft tumors were generated from prospectively consented patients, and their clinical response to platinum-based adjuvant therapy was used to stratify the cohorts of mice bearing serous OvCa xenografts. Tumors collected from patients who did not develop resurgence of their serous OvCa for longer than 6 months were labeled platinum-sensitive. Those tumors derived from patients who developed recurrence <6 months were deemed to be platinum-resistant. Tumor growth in mice was monitored regularly, and treatment regimens were started when tumor volumes were 200–400 mm^3^. Mice were then randomly divided into four cohorts of five to seven mice each. Mice bearing xenografts derived from clinically platinum-sensitive OvCa were randomized to treatment with either MRK-003 alone (300 mg/kg in 0.5% methylcellulose) once weekly by oral gavage, paclitaxel and carboplatin (P/C) alone, MRK-003 with P/C, or vehicles of all three drugs. Mice harboring clinically platinum-resistant ovarian tumors were treated with either MRK-003 alone, paclitaxel alone, MRK-003 with paclitaxel, or vehicles of MRK-003 and paclitaxel. Paclitaxel (15 mg/kg), carboplatin (50 mg/kg), and their appropriate vehicles (Cremophor:ethanol and saline, respectively) were given once weekly by intraperitoneal (i.p.) injection. Tumor volumes were determined every 3–4 days, and mice were weighed weekly. The length of experiments was determined by the individual tumor growth patterns in the vehicle setting so that all arms of experiments could be terminated at one time to decrease biologic variability. At the end of each treatment experiment, mice were euthanized and tumors were harvested. One portion of each tumor was snap frozen, and a second portion was fixed in formaldehyde and embedded in paraffin.

In order to study the acute effect of MRK-003 treatment on Notch pathway activation, mice harboring primary OvCa xenografts were given one dose of MRK-003 or vehicle. Tumors were harvested 6, 24, or 48 h after treatment, and portions were snap frozen as well as fixed in formaldehyde and embedded in paraffin.

### Quantitative PCR analysis

RNA was isolated from frozen xenograft tissue or cell pellets using the GenElute mammalian RNA extraction kit and converted to cDNA (SuperScript VILO, Life Technologies). Quantitative real-time PCR (qPCR) analysis was performed with SsoAdvanced SYBR Green Supermix (Bio-Rad Laboratories) and primers specific for human *Hes1* (forward: 5′-ATTCCTCGTCCCCGGTGGCT-3′; reverse: 5′-TCCAGCTTGGAATGCCGCGAG-3′) and β-actin (forward: 5′- GAGCACAGAGCCTCGCCTTT-3′; reverse: 5′-TCATCATCCATGGTGAGCTGG-3′). For each analyzed sample, relative expression of *Hes1* mRNA normalized to β-*actin* mRNA was determined as previously described (45).

### Statistical analysis

Because of the non-normal distribution of tumor volumes, non-parametric Wilcoxon rank-sum tests were carried out to determine whether the observed differences in tumor volume between the groups in each treatment experiment were statistically significant. All analyses were done using Stata version 11.1 software (StataCorp LP), and a *p*-value of <0.05 was considered statistically significant.

## Results

### Expression of Notch1 and Notch3 in serous ovarian cancer

**Figure 1:**
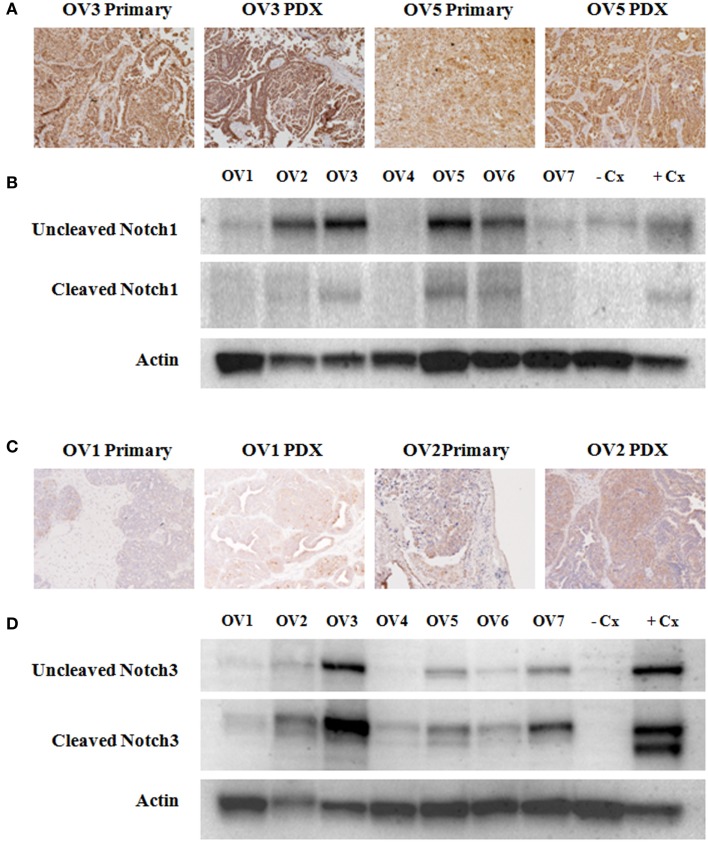
**Variable expression of Notch1 and Notch3 in ovarian cancer specimens**. **(A,C)** Immunohistochemical analyses of Notch1 and Notch3 expression was carried out on xenograft tumors generated from all seven tumors that were subsequently used for treatment studies (OV1–OV7), as well as on paired primary tissue samples when available (OV1–OV5). Representative images of Notch1 **(A)** and Notch3 **(C)** immunostaining are shown. **(B,D)** Western blotting was performed to analyze expression of Notch1 **(B)** and Notch3 **(D)** in xenografts derived from primary human serous ovarian cancers (OV1–OV7). Uncleaved, inactivated Notch1 and Notch3 as well as cleaved activated Notch1 and Notch3 are shown. An anti-Pan-Actin antibody was used to confirm equivalent loading of samples. The HCC1187 and SKOV3 cell lines were used as a negative control (−Cx) for Notch1 and Notch3, respectively. The MCF-7 cell line was used as a positive control (+Cx) for both Notch1 and Notch3.

Notch1 and Notch3 protein expression in both the primary human papillary serous OvCas that were utilized in the current study (OV1–OV7; see Table 1) and in xenografts derived from these patient samples were assessed by IHC (Figures 1A,C). Additionally, Notch1 and Notch3 expression in the same xenografts was analyzed by immunoblotting (Figures 1B,D). Variable expression of both Notch1 and Notch3 was found in all primary tumors and xenografts. IHC revealed that Notch protein levels were consistent between each primary tumor and its corresponding xenograft tumor and largely correlated with the protein level detected by immunoblotting.

**Table 1:** **Clinical characteristics of patients whose tumors were analyzed in this study**.

Patient	Age at diagnosis	Stage	Grade	Progression free	Overall survival	Current status
	(years)			survival (months)	(months)	
OV1	38.3	IV	3	34.1	57.9	Alive
OV2	62.7	IIIC	3	23.9	37.6	Deceased
OV3	60.7	IIIC	3	18.5	24.1	Deceased
OV4	50.8	IIIC	3	22.4	28.9	Alive
OV5	68.4	IIIC	3	7.9	7.9	Deceased
OV6	54.8	III	ND	7.1	10.3	Deceased
OV7	90.5	Unstaged	ND	4.9	6.5	Deceased

### Effect of γ-secretase inhibition on OVCAR3 and SKOV3 cell proliferation and cleaved Notch1 and *Hes1* levels

**Figure 2:**
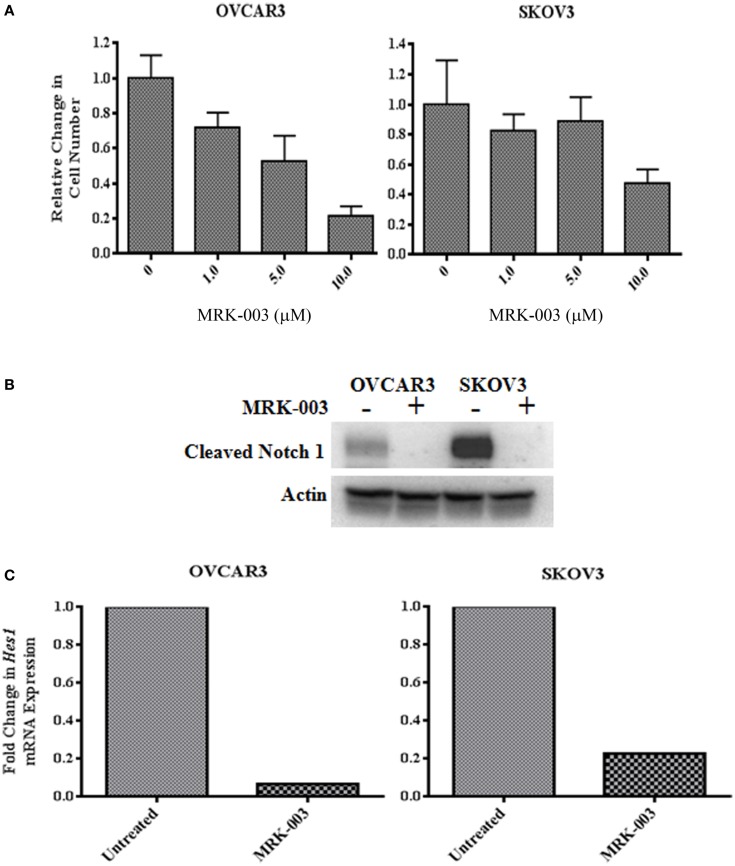
**The γ-secretase inhibitor MRK-003 inhibits OVCAR3 and SKOV3 cell proliferation and decreases NICD1 and *Hes1***. **(A)** *In vitro* treatment of OVCAR3 and SKOV3 cells with MRK-003 leads to a dose-dependent reduction of cell proliferation. Cells were incubated with increasing concentrations (0–10 μM) of MRK-003 for 48 h in medium containing 1% FBS. OVCAR3 cells were treated in triplicate, and SKOV3 cells were treated in quadruplicate. Average relative changes in cell numbers are shown, and error bars represent the standard error of the mean. **(B)** Western blotting analysis of OVCAR3 and SKOV3 cells incubated with 5 μM (OVCAR3) or 10 μM (SKOV3) MRK-003 for 6 h reveals a marked decrease in expression of cleaved Notch1 (Val1744). An anti-Pan-Actin antibody was used to confirm equivalent loading of samples. Control = cells incubated with medium only. **(C)** qPCR analysis of *Hes1* gene expression in MRK-003 treated OVCAR3 and SKOV3 cells, harvested 6 h post treatment, showed significantly decreased *Hes1* transcript levels compared with cells incubated with medium only. Relative changes in *Hes1* mRNA expression are shown, normalized to expression of housekeeping gene β-*actin*.

OVCAR3 and SKOV3 cells were treated with 1, 5, and 10 μM MRK-003. After an incubation period of 48 h, a dose-dependent reduction in OVCAR3 and SKOV3 cell counts was found compared to cells incubated with medium only (see Figure 2A). Subsequently, OVCAR3 and SKOV3 cells were treated with either MRK-003 at the relevant IC_50_ (5 μM for OVCAR3, 10 μM for SKOV3) or vehicle control for 6 h and then harvested. The impact of MRK-003 on Notch signaling was assessed by immunoblotting analysis of γ-secretase-cleaved Notch1 levels and qPCR analysis of target gene *Hes1* expression. We observed both a strong reduction in cleaved Notch1 protein levels as well as a marked decrease in *Hes1* gene expression relative to the vehicle treated control in both cell lines following treatment with MRK-003 (see Figures 2B,C).

### Impact of MRK-003 *in vivo* treatment on *Hes1* expression

**Figure 3:**
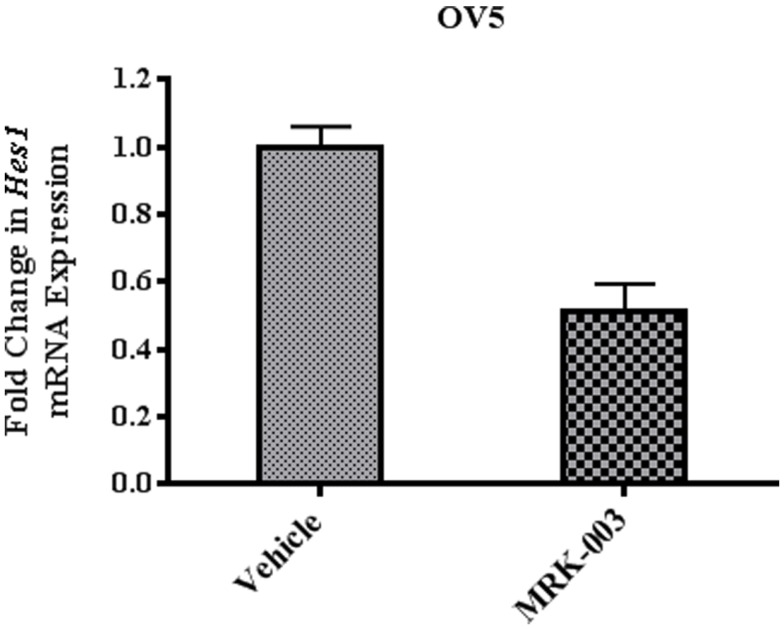
**Treatment with MRK-003 decreases *Hes1* expression *in vivo***. qPCR analysis of *Hes1* gene expression was performed using xenograft samples collected 6, 24, and 48 h after treatment with MRK-003 or vehicle. *Hes1* expression in OV5 xenografts collected 6 h after administration of MRK-003 (*n* = 2) or vehicle (*n* = 2) is shown. Average relative changes in *Hes1* mRNA expression were determined following normalization to β-*actin* expression. Error bars represent the standard error of the mean.

Our *in vitro* data suggest that MRK-003 specifically targets the Notch pathway in OvCa cell lines. To extend these findings, we assessed the effect of MRK-003 on Notch signaling in PDXs. OvCa xenografts were harvested from mice 6, 24, or 48 h after administration of a single dose of MRK-003 or vehicle control and expression of the Notch target gene *Hes1* was analyzed by qPCR. In all analyzed xenografts, a 30–70% decrease in *Hes1* mRNA levels, relative to levels in vehicle treated controls, was observed after exposure to MRK-003. Figure 3 is a representative example of these analyses and illustrates the relative transcript levels of *Hes1* in OV5 xenografts collected 6 h after treatment with MRK-003 or vehicle. OV5 was selected as a representative example as this was a platinum-resistant tumor that demonstrated a potent synergy with paclitaxel in our *in vivo* experiments.

### Assessment of the impact of MRK-003 as a single agent or in combination with cytotoxic chemotherapeutics on ovarian cancer xenografts

**Figure 4:**
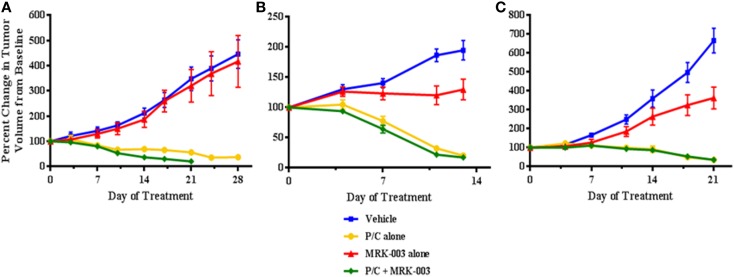
**MRK-003 treatment *in vivo* inhibits tumor growth in the majority of analyzed platinum-sensitive ovarian cancers**. Three independent cohorts of mice bearing xenografts derived from clinically platinum-sensitive serous ovarian cancer were treated with vehicle, MRK-003 alone, paclitaxel/carboplatin (P/C) alone or P/C + MRK-003. A significant anti-tumor effect of treatment with MRK-003 alone was observed in OV3 **(B)** and OV4 **(C)** xenografts (*p* < 0.05). P/C alone and P/C + MRK-003 were equally effective in significantly reducing tumor growth of OV2 **(A)**, OV3, and OV4 xenografts (*p* < 0.01). Tumor volumes were measured twice weekly. Error bars represent the standard error of the mean.

The clinical characteristics of the seven patients whose tumors were used to generate primary xenografts are summarized in Table 1. Briefly, all samples were obtained from women with primarily advanced stage disease, three of whom manifested platinum-sensitive disease and markedly improved survival compared to the platinum-resistant patients. In three independent experiments, mice harboring xenografts derived from a platinum-sensitive tumor were randomized to receive either MRK-003 alone, P/C alone, the combination of MRK-003 and P/C, or vehicle alone. We observed a significant reduction in OV3 (*p* = 0.02) and OV4 (*p* < 0.01) PDX growth following administration of MRK-003 alone, compared with vehicle treated control tumors (see Figure 4). Treatment with P/C alone significantly decreased tumor volumes of all three analyzed platinum-sensitive PDXs, and combination therapy with MRK-003 and P/C was not more effective than treatment with P/C alone (see Figure 4). In Figure 4A, the combined P/C and MRK-003 arm was truncated early so that tumor could be harvested for post treatment investigation. No signs of toxicity were observed in mice receiving MRK-003 and animal weights remained stable during the course of treatment (data not shown).

**Figure 5:**
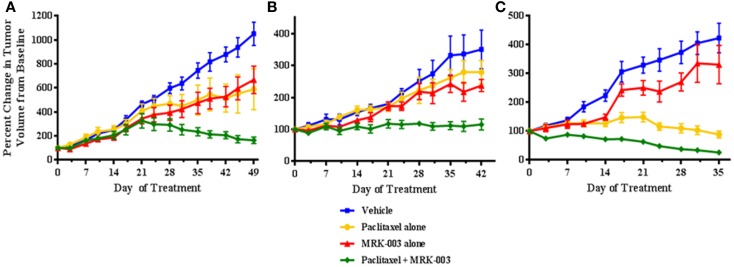
**MRK-003 and paclitaxel synergize to inhibit growth of platinum-resistant ovarian tumors**. Three independent cohorts of mice bearing xenografts derived from clinically platinum-resistant serous ovarian cancer were randomly divided into four groups. Each group received vehicle, paclitaxel alone, MRK-003 alone or paclitaxel + MRK-003. Significantly heightened inhibition of tumor growth was observed upon combination treatment with paclitaxel + MRK-003, compared with treatment with either drug alone (*p* < 0.03). Tumors were measured twice weekly. Error bars represent the standard error of the mean. OV5 **(A)**; OV6 **(B)**; and OV7 **(C)**.

The three primary human platinum-resistant serous ovarian tumors were also established as PDXs in immunocompromised mice. In three separate experiments, mice harboring the tumors were subjected to therapy with either MRK-003 alone, paclitaxel alone, MRK-003 in combination with paclitaxel, or vehicle alone. In all analyzed tumors, enhanced anti-tumor activity was found in mice treated with the combination of MRK-003 and paclitaxel, compared with paclitaxel alone and MRK-003 alone (*p* < 0.03, see Figure 5). Administration of paclitaxel as a single agent led to a significant decrease of xenograft growth in only one of three tumors (OV7, *p* < 0.01, see Figure 5C). In addition, MRK-003 monotherapy led to significant tumor growth reduction of OV5 xenografts, compared with vehicle treatment (*p* = 0.04, see Figure 5A). Again, no significant weight loss or other signs of toxicity were observed in mice treated with MRK-003 (data not shown).

## Discussion

In the current study, we confirm that members of the Notch signaling pathway are expressed in serous OvCa, and GSI-mediated targeting of this pathway leads to anti-proliferative effects *in vitro* and anti-tumor activity *in vivo*. While single agent GSI was tumorstatic in half of the analyzed PDXs, combination therapy with cytotoxic chemotherapy led to significant synergistic anti-tumor effects only in those xenografts originally derived from platinum-resistant OvCa patients. Collectively, these pilot data support the role of Notch signaling in OvCa growth particularly in the chemoresistant setting.

Various studies have detected Notch1 and Notch3 expression in ovarian tumors by IHC, real-time polymerase chain reaction, and/or immunoblotting. The results for Notch1 expression via detection by IHC have been variable and difficult to interpret. In one study of relative Notch1 expression in benign ovarian tissue and a spectrum of low- to high-grade ovarian carcinomas, Notch1 was rarely detected in benign tissue and its expression level correlated with increasing grade and clinical stage of disease (23). Interestingly, Notch1 was primarily confined to the cell membrane and the cytoplasm. The direct functional or clinical significance of increased cytoplasmic expression in OvCa is unclear given that cleaved Notch1 migrates to the nucleus and is considered the active form. In similar immunohistochemical analyses, no nuclear Notch1 expression was detected suggesting little to no role for Notch1 in OvCa (46). In contrast, Notch1 nuclear expression was evident in our cohort of samples but was highly variable among and within samples. Despite the variation between samples, however, the intensity of nuclear Notch1 expression in each primary tumor and its correlate xenografts was stable reinforcing that Notch1 expression remains consistent across subsequent xenograft generations. These data suggest that protein expression of Notch1 or 3 does not correlate with response to GSI suggesting many other molecular inputs must be involved with modulating response in the platinum-resistant setting.

While Notch1 IHC studies have generated conflicting results, immunoblotting analyses that have largely focused on Notch1 NICD expression in primary samples and established human OvCa cell lines are much less controversial. Rose et al. (22) found abundant levels of cleaved Notch1 in the OVCAR3, SKOV3, and CaOV3 cell lines as well as in approximately 75% of the primary OvCa samples analyzed. Consistent with published work (22, 36), we similarly detected cleaved Notch1 in OvCa cell lines and primary ovarian tumor samples by immunoblotting. Thus, in contrast to the collective IHC results, the data to date suggest that cleaved Notch1 is present in some samples thereby providing indirect evidence that Notch1 signaling is active in OvCa.

This hypothesis is further bolstered by functional studies that suggest Notch1 promotes OvCa cell proliferation. In one investigation, stable transfection of A2780 OvCa cells with the Notch1 NICD increased both cell proliferation and the rate of colony formation (26). Others found that down regulation of γ-secretase activity reduced *Notch1* and *Hes1* mRNA and protein in the same A2780 OvCa cells and this decrease correlated with a decrease in cell proliferation (36). Since pan inhibition of γ-secretase likely inhibits activation of all Notch receptors, the observed effects may not be due to specific disruption of Notch1 signaling. Rose and colleagues (22) provided more direct evidence of Notch1 involvement in cell proliferation by siRNA-mediated reduction of Notch1 levels. The decrease in Notch1 in response to siRNA correlated with decreased cell proliferation. Thus, the *in vitro* data suggest that Notch1 signaling can influence the biology of OvCa.

Notch3 has also been implicated in the pathology of OvCa as its expression is evident in a significant number of analyzed OvCa samples. Unlike Notch1, however, investigators have focused on nuclear localization of Notch3 as a functional marker of pathway activation (25, 27, 29, 47). Like Notch1, Notch3 was also expressed at much lower levels in benign ovarian tissue when compared to malignant samples (25, 29), and higher expression levels were associated with either advanced stage (29) or recurrent disease (28, 48). At the genomic level, the *Notch3* gene was amplified in a cohort of OvCa samples, as detected by fluorescence *in situ* hybridization, and this amplification positively correlated with increased Notch3 protein expression (24). This finding was supported by recent data from TCGA, which also showed that Notch3 was amplified in serous ovarian carcinoma (2). Our data support the finding that cleaved Notch3 is present in primary OvCa samples and likely contributes to the pathology of OvCa (24, 25, 39, 48). Blocking Notch3 signaling by a GSI or a Notch3 specific siRNA reduced cell number and led to an increase in apoptosis in OVCAR3 and A2780 OvCa lines (24). Interestingly, the Notch ligand Jagged-1 was abundantly expressed in both OvCa cell lines and benign mesothelial cells derived from tumors (25). Both gene knockdown of Jagged-1 and transfection of the Notch3 intracellular domain (NICD3) suggest the existence of a juxtacrine loop that impacts ovarian cell-cell adhesion and tumor growth (25).

While Notch3 appears to play a role in OvCa cell proliferation, tumor growth, and metastasis, additional investigations have implicated Notch signaling in chemoresistance (28, 39, 48). Knockdown of Notch3 sensitizes OvCar3 cells to carboplatin (28). Additionally, Notch3 is overexpressed in cisplatin and cisplatin/paclitaxel-resistant OvCa cell lines and its inactivation by GSI or siRNA reverses this resistance (48). More recently, OVCA429 cells that were transduced with a retroviral vector expressing NICD3 showed an increase in expression of smooth muscle actin, Slug and Snail, consistent with an epithelial to mesenchymal transition. Interestingly, this shift was associated with an increase in resistance to carboplatin-induced apoptosis (49). McAuliffe and colleagues (39) suggested that tumorigenic OvCa stem cell (CSC) populations defined as CD44^+^ cells or the side population fraction [reviewed by Foster et al. (50)] were decreased with inhibition of γ-secretase activity. These data suggest that Notch signaling may support populations of CSCs in OvCa that are resistant to conventional cytotoxic therapies. Notch signaling can modulate other signaling pathways that may influence CSC function. Specifically, the Notch target *Hes1* modulates *Gli1* expression and Hedgehog signaling providing another mechanism of therapeutic resistance (51). Inhibition of Hedgehog signaling has been shown to augment the effect of P/C as well as delay recurrence of disease in a PDX model of OvCa (44). Given that Notch signaling may support CSC activity in OvCa, inhibition of the Notch pathway could selectively target tumor-initiating populations and act to restore chemosensitivity. This leads to the hypothesis that Notch pathway inhibition in OvCa would produce the most robust effects in a platinum-resistant tumor.

Our investigation suggests a GSI in combination with conventional cytotoxic chemotherapy significantly improves the anti-tumor effects in primary human OvCa xenografts known to be clinically resistant to platinum therapy. This extends the previous findings by McAuliffe and colleagues who demonstrated the efficacy of targeting the Notch pathway in xenografts derived from established OvCa cell lines (39). While this elegant study demonstrated GSI induced anti-tumor activity restored chemosensitivity, it was unclear if primary-derived ovarian tumors would respond to therapy *in vivo* in the same fashion. From a clinical perspective, platinum sensitivity, defined as a >6 month progression-free interval after platinum-based therapy, is one of the most important prognostic factors for women with OvCa (52, 53). In order to better understand the *in vivo* response to GSI, we utilized PDXs derived from women with known platinum-sensitive or platinum-resistant disease. We observed that single agent MRK-003 significantly inhibited tumor growth of two of three platinum-sensitive ovarian tumors, as well as one out of three platinum-resistant tumors. When we combined GSI with conventional cytotoxic therapy, synergistic activity occurred only in the clinically defined platinum-resistant tumors. These findings support data showing that inhibition of Notch signaling increases sensitivity to chemotherapy (32, 39), implying that the Notch pathway is involved in the development of chemoresistance.

Currently, early phase clinical trials assessing treatment efficacy of either single agent GSI or monoclonal antibodies focused on disruption of ligand binding have been conducted in a number of solid tumors including thyroid, non-small cell lung, desmoids, and OvCas (54). The overall promising efficacy of disrupting Notch signaling has been tempered by some adverse events, which are primarily gastrointestinal (55, 56). In our study, we used the pan GSI MRK-003 whose clinical analog MK-0752 is in development for treatment of several solid malignancies (56). Although treatment with other GSIs has resulted in toxicity, we did not observe any excessive weight loss or treatment related death in our experiments.

The success of targeted therapies inhibiting Notch signaling will also depend upon the development of biomarkers that can identify those patients who are most likely to respond (54). Currently, there is no universal biomarker that indicates Notch family dependence and is correlated with response to a particular GSI. This objective may become more complicated given recent evidence that Notch signaling can serve an oncogenic or tumor suppressor function depending on the disease context (57). We observed no correlation between baseline Notch1 or Notch3 protein levels and response to treatment with MRK-003 alone in any of the ovarian tumors we analyzed. A marked decrease in *Hes1* mRNA expression was observed in both the platinum-sensitive and platinum-resistant PDXs following GSI therapy (data not shown), confirming that this downstream marker of on target effect is also of limited value for associating anti-tumor effect. Recent work examining the efficacy of MRK-003 in pancreatic cancer xenografts similarly did not identify a clear biomarker (9).

In conclusion, this pilot investigation suggests specific Notch family members are variably expressed in OvCa and that MRK-003-mediated inhibition of Notch signaling reduces OvCa cell proliferation *in vitro* and inhibits tumor growth *in vivo*. While single agent anti-tumor activity was observed in many of the PDX analyses, GSI improved conventional cytotoxic therapy exclusively in combination with paclitaxel in those OvCa tumors known to be clinically platinum-resistant. Our results support the rationale for further investigation of the pre-clinical and clinical effectiveness of GSIs in OvCa, especially in the platinum-resistant setting.

## Author Contributions

Jolijn W. Groeneweg: study design, experimental conduct, data analysis, and manuscript preparation. Celeste M. DiGloria: sample procurement and experimental conduct. Jing Yuan: target validation. William S. Richardson: experimental conduct. Whitfield B. Growdon: study design, data analysis, and manuscript preparation. Sriram Sathyanarayanan: target validation and manuscript editing. Rosemary Foster: study design, data analysis, and manuscript preparation. Bo R. Rueda: study design, data analysis, and manuscript preparation.

## Conflict of Interest Statement

Sriram Sathyanarayanan was an employee at Merck Oncology, Boston, MA, USA at the time this study was designed and initiated. All other authors reported no conflict of interest with the work presented in this manuscript.
